# Co-Designing Communication: A Design Thinking Approach Applied to Radon Health Communication

**DOI:** 10.3390/ijerph20064965

**Published:** 2023-03-11

**Authors:** Sofie Apers, Heidi Vandebosch, Tanja Perko, Nadja Železnik

**Affiliations:** 1 Department of Communication Studies, University of Antwerp, 2000 Antwerp, Belgium; heidi.vandebosch@uantwerpen.be; 2 Institute for Environment, Health, and Safety, Belgian Nuclear Research Centre, SCK CEN, 2400 Mol, Belgium; tanja.perko@sckcen.be; 3 Department of Political Science, University of Antwerp, 2000 Antwerp, Belgium; 4 Elektroinštitut Milan Vidmar, 1000 Ljubljana, Slovenia; nadja.zeleznik@eimv.si

**Keywords:** radon, persuasive communication, participatory design, co-design, health intervention

## Abstract

Indoor radon is a natural radioactive gas and is one of the leading causes of lung cancer. Despite multiple policy and communication interventions to increase radon testing and mitigation, the uptake of these measures remains insufficient. A participatory research design was applied in Belgium and Slovenia to probe the barriers and facilitators homeowners experience regarding radon protective behavior on the one hand and co-designing communication tools on the other hand. The results show that there remains a need for interventions on all levels (i.e., policy, economic interventions, and communication). Moreover, results indicated a need for a communication strategy that follows the different steps between awareness and performing mitigation measures. Further, involving the target group in the early stages of intervention design was beneficial. Future research is needed to test the effectiveness of the proposed communication strategies in a controlled setting.

## 1. Introduction

Health intervention planning models emphasize the importance of participatory methods, thus involving community members and other relevant stakeholders in the different planning stages, from problem definition to intervention implementation [1]. Not only does this increase the external validity of the intervention by the acceptance and acknowledgment of the input provided by the community, but it also provides broad perspectives and skills from community members, stakeholders, and the design team. Using the collective creativity of professionals and the local community in designing an intervention is referred to as co-design [2] and can be seen as a citizen science approach [3]. Although multiple citizen science projects were conducted within the field of radon, co-design methods have, to our knowledge, not yet been adopted in intervention design [4].

Radon is an indoor air pollutant. It is a natural radioactive gas that is present in the soil in varying concentrations depending on the composition of the ground. Radon is invisible and has no scent, there are no visible casualties due to the gas, and since it is a natural gas, there is no culprit to blame [5]. In high-risk areas, radon can enter houses through cracks or different installation tubes in the foundations of buildings, and the gas can accumulate indoors. Radon concentrations are one of the leading causes of lung cancer [6].

Despite current health interventions, research shows that testing and mitigation rates remain insufficient [7,8]. This raises the question of whether the current interventions tackle the right barriers and provide the right facilitators. Research specifically focused on (mass) communication interventions regarding radon has observed multiple gaps in the communication strategies adopted in the past. For instance, statistical information in leaflets or news articles prevails [9].

To address these gaps, an exploratory co-design study was developed to first focus on general barriers and facilitators to perform radon protective behaviors and second on the ideation and designing of communication interventions, together with people with personal experience with radon. In this way, community members co-design a communication intervention, making it more personally relevant and likely more effective [1].

## 2. Literature Review

### 2.1. Health Interventions to Address Radon Exposure

Changing behavior requires change on different levels; the behavior change wheel identifies capability, opportunity, and motivation as the main sources of behavior. Motivation reflects the individual, opportunity reflects the individual’s environment, and capability reflects a combination of the two. For behavior change to be effective and durable, the three components should be addressed with different types of interventions that often stem from the policy level [10].

Looking at the policy level regarding radon, Europe adapted the Basic Safety Standards in 2013 and included radon protection as well [11]. In practice, all European Member States are legally required to develop and implement a radon action plan containing information on ways to decrease radon levels at homes and workplaces. In the United States, the Indoor Radon Abatement Act (IRAA) from 1988 requires that indoor radon levels be as low as outdoors [12]. These legislations, however, are on the highest level (namely the European level and the National level of the United States). The responsibility lies with the countries/states and their interpretation of their responsibility and legislation. Some countries/states, for instance, Estonia, only inform people about radon and place the responsibility for behavioral actions on the individual [13], whereas other countries, for instance, Ireland and Belgium, take the initial steps to include more specific legislation [13,14]. Multiple scholars state that legislation procedures in terms of housing code requirements (comparable to energy efficiency) might increase the uptake for radon testing and mitigating [15,16,17], as is the case in certain States in The United States, for instance, Pennsylvania [12]. On a European level, Austria is considering similar measures [13].

Other policy measures are mostly concerned with reducing the economic impact of the testing and mitigating procedure—for instance, incentivizing mitigations, offering subventions, or providing free tests [15,17]. A city in Ireland experimented with providing digital radon monitors in the library to facilitate the need for these monitors without the costs of buying them [3]. Other countries, such as Bulgaria and the Czech Republic, provide free tests, and yet other countries (e.g., Belgium) sell tests at lowered prices during the heating season. Subventions for mitigation are also country-dependent; for instance, Austria, Germany, and Sweden provide financial support to those carrying out mitigation works [13]. No real evidence is available on whether the financial aspect matters to people. Interestingly, focus groups in Ireland show that people who performed mitigation perceived the costs as not too high as it was an investment in their health. At the same time, people who did not mitigate (but had high levels of radon) perceived the costs as too high and an important barrier [18].

Despite the interventions and measures in place, the uptake of radon protective behavior remains insufficient [7]. It remains unclear whether the interventions in place address the barriers people experience and whether they create the right facilitating conditions. Therefore, there is a need to explore in more depth what barriers and facilitators people experience regarding radon-protective behavior.

As radon is a multi-level problem, not only do the situational and the environmental factors matter, the responsibility of actually performing testing and mitigating often still lies with the individual homeowners [19]. So, while creating the right environment for them to act is needed, they still must be motivated to act. One way to increase motivation is through communication and persuasion. Communication occurs on different levels, including interpersonal communication (e.g., an individual talking about radon with their general practitioner), stakeholder communication (e.g., general practitioners that are informed about radon on a higher level), and mass media communication (e.g., press articles about radon).

A recent systematic review that focused on mass media communication about radon [9] shows that campaigns mostly aim to increase awareness, knowledge, risk perception, and perceived susceptibility using factual communication in the form of brochures or press articles. The focus is on providing people with information about the characteristics of radon and the (technical) solutions. Although informative leaflets can be effective, they assume the full rationality of the audience, where they act upon the information they receive. The literature on behavior change has shown that people often experience bounded rationality and that other aspects, such as relevance, biases, and emotions, play an important part in the process [20]. Other messages such as fear appeals in videos showed increased intention to request more information [21], and direct phone calls and letters increased intention to test [22].

Moreover, while these communication interventions have shown to be effective to some level (e.g., low degree of increase in testing behavior), the next step, namely mitigation, remains mainly unchanged [9], which identifies an additional gap. In particular, Hevey identified 17 steps of behavior, from becoming informed about radon to having confirmed mitigation [20]. However, communication interventions rarely move along these steps. The precaution adoption process model is a theory based on the different stages of behavior, from being unaware of the problem to maintaining the problem. The theory emphasizes that different stages require different communication approaches. For instance, to move from the first stage (unaware) to the second stage (unengaged), media messages about the hazards are needed, while in progressing from the second stage to the third (undecided), testimonials and personal experiences are most effective. Further, to proceed from the third stage to the fourth (decided not to act) or to the fifth (decided to act), information about personal susceptibility, likelihood, and severity of radon exposure is effective. Detailed information about ways to perform the behavior, the costs, and the resources are mainly effective when moving from the fifth stage to the final stage (maintenance) [1,23,24,25].

Overall, the systematic review showed a need for more personally relevant communication efforts, as the question remains whether and to what extent the current communication approaches tackle the right determinants at the right moment and are in line with the needs of the public [9]. This unveils the need to inquire about the their preferences of the target group regarding radon-related communication.

### 2.2. Co-Design in Health Interventions on Radon

To answer these questions, we need to engage in dialogue with the target group themselves and, even more so, involve them actively in developing communication tools. Participatory designs include various methods; however, the mean denominator is the active engagement of the public. Different levels exist within participatory designs, from providing information (one-way) to a discussion (two-way) and active participation (multiple ways), which is the highest level of involvement. The latter often results in participatory decision-making and co-design of new products, technologies, or health interventions [26].

Within the existing research about the health issues related to radon, participatory designs or citizen science projects have been adopted previously [3,4]. The main topic investigated in previous studies was the understanding of the lack of mitigating behavior, either through interviews (i.e., providing the information) [15,27] or through discussing the topic in focus groups (i.e., discussion) [28,29]. Citizen science projects were related to, for instance, raising awareness, radon mapping, or radon testing and mitigating [3]. To our knowledge, ours is the first study applying active participation in the design process of a communication intervention in the context of radon.

More specifically, our study was designed to involve residents and homeowners in understanding the lack of radon protective behaviors and related general barriers and facilitators and considering solutions regarding communication campaigns. To investigate these aspects, we opted for design thinking. This participatory design framework allows for opening up the problem and inviting people to think along to identify it and create solutions based on their first-hand experiences [30]. It is a way of creative problem-solving that is human-centered and emphasizes observation, collaboration, and visualization of ideas. It emphasizes empathizing with the issue and the context of the issue, defining the exact problem and challenge, ideating ways to solve the challenge, and testing prototypes to do so [31]. This method, both problem- and solution-oriented, can provide new insights into why people avoid radon protective behaviors, what they think the solution would be, and even what the solution should look like.

To summarize, two questions are raised: first, what are the main barriers and facilitators to engaging in radon-protective behavior experienced by homeowners, and how are these addressed in current interventions, if at all? Second, how can the communication about radon be improved to be more relevant and engaging for the target group?

## 3. Materials and Methods

To apply the participatory design, we composed a research team comprising researchers from different disciplines, such as risk communication, health communication, sociology, nuclear physics, and citizen science. This ensured the avoidance of conceptual bias. Most researchers of the team had expertise with qualitative methods and radon research; however, none had operational expertise in design thinking as a research method. Therefore, the research protocol was developed in collaboration with a Belgian company specializing in design thinking (ACOMPANY). The company also provided a full training day of the method for all researchers involved in this study.

### 3.1. Participants

The aim was to recruit participants who already had some experience with radon so that they could speak from their own experiences rather than a hypothetical scenario. This meant that we recruited people who had already measured (high) radon levels.

### 3.2. Workshop Design

**Figure 1:**
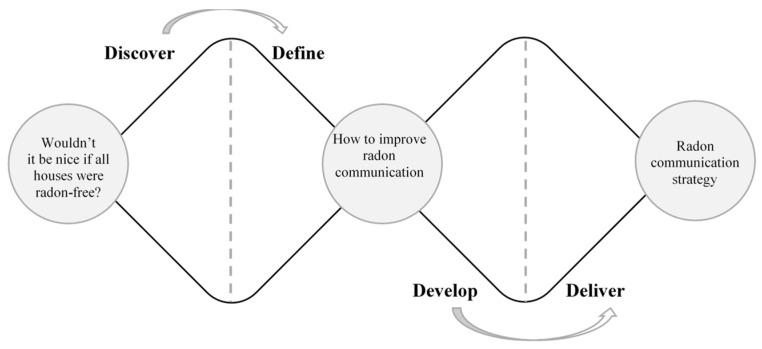
The double diamond framework applied to radon.

A workshop was designed that consisted of two unstructured group sessions. Each session lasted two hours and was scheduled a week apart. More specifically, the framework of the double diamond was applied to the context of radon and the workshop design itself [32]. The first stage of this framework, as seen in Figure 1, is the challenge, which is the starting point of the workshops and describes the ideal scenario. For this research project, the challenge was defined as “would it not be nice if all houses were radon-free,” referring to the ideal scenario where radon protective behavior is performed and facilitated easily among all homeowners in radon-prone areas. In the first session, the participants used this challenge to consider why houses are not already radon-free. In other words, “would it not be nice if all houses were radon-free” was the initial prompt to discuss barriers and facilitators in the first session. Since the participants all had experience with radon, this prompt was understandable for the participants as a starting point.

Participants recorded all the problems (i.e., barriers) that arose on post-it notes while discussing them. These problem statements could relate to the causes of the challenge, the importance, the target audience, and other related issues, specifically in the form of “how-to questions.” This stems from the concept of *how to* ensure that *all houses are radon-free,* formulating a barrier as a facilitator; for instance, “how to make people aware” (i.e., facilitator) refers to the lack of awareness (i.e., barrier). Once saturation was reached and no new problems were added, dot-voting allowed for defining the most pressing problem statements. In other words, the first session discovered *the why* of the main challenge.

Between the first and second sessions, the problem was defined further. In this case, the problem definition for the second session was “how to improve radon communication.” In the workshop’s second session, this was used as the prompt to start the discussion, together with the main findings from the first session. In this session, the focus was on ideation and brainstorming. The participants discussed potential radon communication strategies, selected the ones they considered the best, and started to develop protocols for the materials, which led to a communication strategy. This session explored *the how* of the main challenge. Both sessions aimed to diverge first (i.e., creating options) and converge afterward (i.e., selecting options).

One of the tools often used in design thinking approaches is developing a customer journey, which indicates all the steps between being aware and purchasing a product or even becoming an ambassador (i.e., as a customer actively promoting the product among peers). Based on the precaution adoption process model [24] and the 17 steps of radon behavior developed by Hevey [20], a homeowner journey was developed before the workshops. Seven steps were identified: awareness, evaluation of the knowledge (i.e., engagement with the health issue), purchase of radon test kit, delivery and conducting radon test, action (i.e., mitigating home), reassuring (i.e., confirming successful mitigation by re-testing), and ambassadorship (i.e., convincing others about the importance of radon tests). For every step, barriers, motivations, emotional states, and actions were identified. Developing the homeowner journey ensured a complete overview of the available literature about radon behavior. The full homeowner journey can be found in Appendix A. If the discussion dtalled, the homeowner journey was an additional prompt during the first sessions.

The workshops were conducted in Belgium and Slovenia.

### 3.3. Workshop 1: Belgium

Effects of radon are a significant health problem in Belgium. Approximately 48% of the Walloon region in Belgium is expected to be affected by radon [33]. Radon likely contributes to approximately 480 deaths due to lung cancer per year [34]. To prevent this, approximately 36.000 dwellings need to be mitigated [14,35].

The Federal Agency of Nuclear Control (FANC) is responsible for organizing activities to apply the regulations, comply with the obligations, and raise awareness of the actors involved in radon. Therefore, FANC strives for close collaboration with multiple actors, such as the provinces, municipalities, professional organizations, academic institutions, and the public. While exposure to radon at work is regulated and the employer is responsible for mitigating the working place, mitigation of dwellings is not legally required. It remains the responsibility of the homeowner [14].

To increase the number of radon tests in dwellings, regional authorities contribute to radon test kits, which means that the price for a test kit is reduced from 30 euros to 15 euros. Financial help from the regional government for mitigation actions is also in place. The mitigation of a dwelling in Belgium costs between 500 euros and 5000 euros. Lists of companies with expertise in radon mitigation are published online [35].

A communication plan was defined in 2014 and is updated yearly based on the evaluation of the past year to improve awareness and increase mitigation rates. In this context, a dedicated internet page was established. The effectiveness of the communication interventions is evaluated for the most impactful activities, such as orders of test kits. Other measures such as reach (e.g., visits to internet pages) and media return are also evaluated. FANC also tested social advertising in 2021 (paid ads on Twitter). However, this campaign was not further evaluated. The results of a public opinion survey show that 32% of the population are aware of radon and that 11% of them applied some mitigation measure in their home [36].

The first workshop was conducted in March 2022 in Belgium. Due to COVID-19 restrictions, both sessions occurred online. An online whiteboard was used as an online alternative to physical post-its.

#### 3.3.1. Sample

Recruitment was conducted through local authorities, who spread the message about the workshops on their social media and websites. The principal investigator also contacted radon mitigation companies, who, in turn, forwarded the message to people that had completed (or were in the process of completing) radon mitigation. This way, people were invited to contact the research team to enroll in the workshops.

The sample of the first workshop consisted of six participants, of which four detected radon in their homes, and two were professionally engaged with radon. Three participants belonged to the same family, all living in Luxembourg. This was unforeseen and only known at the start of the first session, but due to recruitment challenges, we decided that they still could participate as their experiences could inform us as well. In every session, five participants were present, with four overlapping participants in both sessions.

#### 3.3.2. Facilitation

Facilitators of ACOMPANY moderated the workshop in Belgium. This allowed the research team to observe and learn the methods they adopted. During both sessions, the researchers observed without interfering, as the objective was to explore first-hand barriers and solutions of the participants. This workshop demonstrated some limitations to the online format; therefore, we decided to wait until the end of COVID-19 restrictions to host the second workshop face-to-face.

### 3.4. Workshop 2: Slovenia

Due to its geology, Slovenia has many municipalities heavily influenced by radon. It is estimated that 100 people per year die due to lung cancer caused by radon [37]. To prevent radon-related deaths, the Slovenian Radiation Protection Administration is responsible for the Radon Action Plan [38]. Through online and face-to-face meetings, it consults with all ministries involved with radon, including the Ministry of Health and Ministry of Environment, Technical Support Organizations, and Education. Free measurements for dwellings are available for residents in radon-risk areas; however, the number of available tests is limited. The average mitigation costs for standard dwelling amount to approximately a few thousand euros. Target groups of communication interventions are employers, employees, local decision-makers, and the public in general. Communication interventions are focused on increasing awareness and are mainly developed in the form of brochures. Other strategies include news articles, seminars, expert meetings, workshops, and a comic book for children [39]. Perko and Turcanu determined that the frequency of personal advice, dialogue, and response to radon-related questions and concerns of residents are very good in Slovenia compared to other European countries [40]. The effectiveness of the communication interventions is not measured, and objective radon awareness measurements among residents are unavailable.

In May 2022, the second workshop occurred face-to-face in Slovenia. The recruitment was also conducted through local authorities; however, it was also picked up by local media, such as the local radio and newspaper.

#### 3.4.1. Sample

The sample of the second workshop consisted of 9 participants for the first session and 8 participants in the second session. All of them were residents from a high-risk area in Slovenia who were experienced with testing their homes and detected indoor radon concentrations above the reference level of 300 Becquerel/m^3^. They all were either planning to mitigate or had already performed mitigation measures.

#### 3.4.2. Facilitation

The second workshop was moderated by two researchers of the research team, native Slovenian speakers with experience with moderating qualitative research. The researchers who conducted the second workshop were briefed by those who observed the first one to align the workshop procedures.

### 3.5. Data Analysis

Both workshops were recorded and transcribed according to the ethical guidelines of the social sciences. The research team conducted an inductive thematic analysis, adopting a semantical approach. The participants recorded their main thoughts regarding the barriers, facilitators, and communication approaches on post-it notes. Therefore, their views, opinions, and experiences were made explicit, hence the semantic approach. These post-it notes were used to code the transcripts to provide more background information. After each session, these post-it notes (i.e., codes) were categorized thematically by the research team, until a consensus was reached. Since the approach was to explore the barriers, facilitators, and communication ideas, no pre-defined codebook was used.

## 4. Results

### 4.1. Workshop 1: Belgium (Online)

#### 4.1.1. Session 1: Problem Statements

The results of the first session were oriented toward problem formulations related to the following challenge: “would it not be nice if all houses were radon-free?”. In total, 36 problem statements were formulated, identifying the underlying barriers and facilitators. Not all of them were in the “how-to” format. However, they were still valuable in emphasizing certain problem areas. The following are examples of problem statements: “How to establish an EU standard?”, “How to oblige radon measures in new buildings?”, “How to find help from the state?”, “How to facilitate the necessary steps?”, “How to shock people?”, “How to develop a decision tree? ”, etc. The full list of problem statements can be found in Appendix B.

Another example includes problem statements such as “How to make people aware?”, “How to ‘touch’ people?”, “How to visualize the danger?”:

*“… we realize that people don’t know about radon in our country. I live in the province of Luxembourg [Belgium], which is the most affected. And despite everything we do, people don’t know about it. I think that if we want to be able to act and do something, people must first know.”*

(P2)

*“One difficulty is that when we talk about the FANC [Federal Agency of Nuclear Control], we don’t know, it’s something we don’t know too much about, which is, which is not close to here. So, there is a certain distance, both physical and perhaps also in the consciousness of people.”*

(P3)

Other problem statements included issues related to “How to get help to remediate?”, “How to find reliable information?” and “How to find the right solution for the right house?”:

*“To give you an example, we have a list of companies in Luxembourg [country] that should be able to deal with radon. We contacted them all, the whole list, there is nobody who really has experience on it, but they are on the list of experts.”*

(P5)

After diverging, i.e., collecting different problem statements, and after saturation was reached, the participants converged by choosing the problems that they felt were most important, as presented in Table 1. Participants compiled their top 3 issues. To provide an overview of the prioritized issues, researchers attributed 3 points to their number 1, 2 points to their number 2, and 1 point to their number 3. The ones with the most points are therefore considered the most important.

**Problem definition**

After the first session, researchers clustered the problem statements thematically to identify the underlying facilitators. The following categories were formulated: installing standardization to ensure quality (*n* = 7), clarifying a stepwise approach (*n* = 4), communication through different stakeholders (*n* = 4), thresholds (*n* = 7), cost of mitigation (*n* = 2), mitigation contractors (*n* = 2), and communication (*n* = 10). The full overview can be found in Appendix B. Since the study aimed to co-design communication tools, the problem definition was also related to communication. Since communication was also highly represented and comprised some of the prioritized problem statements, this decision was justified.

#### 4.1.2. Session 2: Solution Statements

In the second session, the working statement concerned communication. In total, 41 ideas were presented by the participants. Examples of ideas are workshops in primary schools, including general practitioners in the communication concerning radon, creating a “radon safe” label, a testimonial of someone who easily mitigated, a catchy radio spot with humor, advertising via social media, more visibility to mitigation companies, flyers in public spaces, etc. The full list of communication ideas can be found in Appendix B.

After saturation during the brainstorming, participants converged by voting for their favorite ideas. They each had two votes, and the results are presented in Table 2.

During this session, the facilitator prompted ideas for four steps of the homeowner journey: radon awareness, evaluation (before testing), action (i.e., mitigation), and ambassadorships. To simplify the process for the participants, the research team decided to map the ideas to the homeowner journey among themselves after the session. Some ideas were mapped in multiple stages. The full overview can be found in Appendix B.

Most of the ideas were mapped to the first (*n* = 20) and the second step (*n* = 20) with a lot of overlapping communication strategies such as an advertising campaign via social media, a catchy radio spot with humor, a booklet in schools, press articles and flyers. In the action step, fewer ideas were presented (*n* = 14), and these strategies implied more specific information. Examples include a testimonial of someone who easily mitigated radon effects, flyers with information about mitigation costs, showing examples of other people who mitigated, showing pictures that emphasize the simplicity of the process, and providing more visibility to solutions and mitigators. Finally, the last step, ambassadorship, was the one with the least ideas (*n* = 5); however, those ideas do emphasize the social component of communication strategies, including, for instance, an advertising campaign on social media, a testimonial, creating a “radon safe” label, or organizing a competition with prizes for people who mitigated their houses.

Due to the limits of the online format in time management and lacking group dynamics, the second session of the first workshop ended with prioritizing solutions and did not further proceed with designing the solutions.

### 4.2. Workshop 2: Slovenia (Face-to-Face)

#### 4.2.1. Session 1: Problem Statements

Similar to the first workshop in Belgium, the first session in Slovenia was oriented toward problem formulations; however, the highly involved participants had already started formulating solutions at this stage. Despite the different formats, the solutions provided in this first session also expose underlying issues. For clarification, we rephrased the solutions from the first workshop to problem statements; however, the original formulations can still be found in Appendix C. In total, 45 problem statements/solutions were formulated. A few examples include: “How to include radon as a topic in schools?”, “How to provide understandable and accessible information about mitigation?”, “How to provide accessible free dosimeters?”, “How to get subventions from the state?” “How to guarantee the quality of the mitigation works?”, etc. The full list can be found in Appendix C.

Another example is “How to increase awareness about radon in the population?”. Multiple participants indicated that they learned about radon through their social networks:

*“Well, then one of my friends was encouraged [to test], and she also said, I didn’t know either, I didn’t know, and the problem is that we ordinary people don’t even know, unless we are really terribly interested in it, to even report it so that you can measure it.”*

(P6)

*“We had a measurement done because a friend of ours had done it a couple of 500 m away, and then we had it done.”*

(P9)

After diverging, and when no new problems were added, the participants converged by voting for the most important problem statements in their opinion. They each cast three votes. The issues with the most votes were the most important barriers. The results of the dot voting can be found in Table 3.

**Problem definition**

The problem statements were clustered thematically by the researchers, resulting in the following categories: communication, information, and awareness (*n* = 10), advice after measurement (*n* = 6), comprehensive/holistic approach (*n* = 3), accessibility of passive and active dosimeters and measurement support (*n* = 9), mitigation support (*n* = 5), the financial burden of mitigation (*n* = 5), the legal requirement (*n* = 6), and motivation (*n* = 1). The full overview can be found in Appendix C. Similar to the Belgian workshop, the communication, information, and awareness category were emphasized. Again, this justified the decision to focus on communication in the second session. More specifically, the following questions were raised: How do you think radon awareness should be raised? Moreover, how should advice on mitigation be communicated?

#### 4.2.2. Session 2: Solution Statements

For the first question about awareness, 22 ideas were formulated, including advertisements on YouTube, TikTok, and Instagram, regular information about radon in mass media, personal letters to all households, an interactive portal about radon, radon education in schools, contributions about radon in TV, radio, and newspapers. The participants voted for the best ideas, which can be found in Table 4.

The group then discussed the details of the personal letter (i.e., informing households by post). For instance, the participants discussed that the letter should cover the prevalence of radon, the dangers, locations and ways to order dosimeters, the concerning radon values, and an invitation to participate in the measurements. They discussed that the municipality should draft the letter with an official signature. Further, they discussed the possibility of opening a special office to manage the radon campaign. The group also discussed whom to target and whether it should be addressed or unaddressed mail. They mentioned that a special message could be printed on the envelope, such as “it’s about your health.” The participants agreed that the letter should be sent in the winter. Creating a logo or corporate identity was also discussed, using red and yellow, as these colors are associated with radon areas, and green because it is associated with a solution. In the first part, the logo should be intimidating, and reassuring in the second part, as a solution. The group also discussed that the letter should be distributed by e-mail and social media.

For the second question concerning the advice on mitigation, the group formulated 13 ideas. Examples included personal testimonials of people during mitigation, a list of mitigation contractors, social media campaigns, and personal communication with a selected advisor. The full list can be found in Appendix C.

Results of voting for the second question, resulting in the following prioritized ideas, can be seen in Table 5.

The idea that received the most support was to hear people’s testimonials about their experiences with mitigation. The stories could either include a successful experience or lessons learned from less successful experiences. There was an idea to organize this through social networks online, for instance, through municipalities on social media. The group agreed that the information should not be too technical and should not resemble a commercial. Finally, they also discussed the need to target younger generations who are buying and building houses, and that information channels should be chosen accordingly.

## 5. Discussion

By setting up a qualitative co-design workshop with homeowners, we aimed at gaining more in-depth knowledge about the barriers that people experience in mitigating their house, on the one hand, and collecting their creative input and insights about ways to communicate the dangers of radon could be improved on the other.

First of all, the results show that the barriers people experience are situated within different levels of interventions and different steps of behavior, as described in the literature review. The stages discussed in this section are simplified and focus on awareness, testing, and mitigating behavior for clarification purposes.

Barriers related to the first stages of behavior were focused on a lack of awareness and engaging communication. The participants agreed that awareness should be the first step. In Belgium, the focus was placed on more attention-grabbing awareness campaigns, such as social media campaigns and humor, while Slovenia focused on personalized letters. This is in line with the research of Weinstein et al., where they tested whether personalized phone calls and letters affected perceived susceptibility and self-protective behavior (i.e., intention to test). They determined that personal susceptibility did increase significantly for those who received the phone call and the letter; however, no differences were detected in terms of intention to test. This could indicate that the proposed letters by the participants could successfully increase engagement with the health topic, yet that other communication strategies are needed to address the further steps in the mitigating process [41]. These results also show the nuance of the concept of awareness, where a discrepancy between being aware and making a personal risk assessment remains. As Poortinga et al. [42] reported, high levels of awareness do not always result in higher levels of concern; therefore, raising awareness could be focused more on grabbing attention and raising curiosity rather than merely informing.

Barriers associated with testing behavior include the lack of available active and passive dosimeters in Slovenia. According to the participants, communication in this stage should be more specific than in the awareness stage; for instance, a comprehensive website with information, workshops, or newspaper articles would provide them with the information they need without overwhelming. Moreover, information from different stakeholders, such as medical doctors, could help emphasize the importance of radon testing. Apart from the accessibility of tests in Slovenia, no issues were mentioned regarding the costs of test kits.

When examining the next stage, it can be observed that many barriers are related to mitigating behavior. Participants highlighted the importance of personalized advice after testing, with a clear step-wise approach on what steps to take next and how to do so. Finding mitigating companies with radon experience was challenging, according to the participants in Belgium and Slovenia. Moreover, the lack of guaranteed results after mitigation was a particularly important barrier in Belgium. Participants indicated that this had to be the state’s responsibility to implement regulations for these companies, as that would facilitate the process of the homeowners finding the best help for their particular radon problem. This could be achieved by certifying certain mitigating companies or involving inspections at mitigating companies, as proposed by the participants. Further, the financial burden of mitigation was mentioned in both workshops, emphasizing the need for subventions or financial aid from the government.

Regarding mitigation behavior, the participants indicated a need for communication on different levels, for instance, stakeholder communication. They felt the involved stakeholders (e.g., medical professionals, mitigating companies, local authorities) are not sufficiently up to date in helping homeowners accordingly with radon issues. Especially in this stage, participants expressed a need for detailed and clear information, and both countries suggested using testimonials. The participants emphasized that the testimonial should contain a story of someone who mitigated their house or what lessons could be learned from unsuccessful mitigations. In that way, both the problem and the solution were addressed. This idea is already supported by the literature on narratives, stating that narratives could help in facilitating information processing, comprehension, and recall [43].

Overarching barriers were related to legislation and regulation. On the policy level, both workshops showed a need for obligatory radon measures in new buildings; moreover, a need for a European standard was also expressed in Belgium. Despite the European Basic Safety Standards and the inclusion of radon measures in the building permit in Belgium, participants still expressed these aspects as a need for future policy-level interventions (Council Directive 2013/59/EURATOM, of 5 December 2013). This aligns with the current policy measures; however, the policy must be implemented sufficiently to impact homeowners’ barriers. Further, policy changes in adding radon levels to the energy certificate to regulate radon levels in the housing market were proposed, which agrees with previous research on mitigating [15,16,17].

Regarding communication, the participants highlighted the need for a holistic step-wise approach, where communication follows the different stages of behavior and a consistent message is conveyed across stakeholders, channels, and time. Generally, it is important to note that behavior change will only occur if the environment is ready. In other words, barriers related to, for instance, the availability of dosimeters and mitigation companies should be addressed first before communicating about the health risks to ensure fitting solutions are available.

This study indicated that co-design workshops and participatory research are crucial to gaining the users’ perspectives and ideas early in the intervention design. The face-to-face workshop was preferred when comparing both workshops, especially since this setting increased the group dynamic and collaboration efforts. The online format was, given the circumstances, still valuable in understanding the barriers and collaborating on communication ideas, yet a face-to-face setting was needed to conduct an even more in-depth inquiry. Design thinking workshops have shown to be valuable in the intervention design process related to radon; however, other health topics could and should also be addressed with participatory methods, such as design thinking, early on to maximize the involvement and input of the target group.

### 5.1. Limitations

Just like any study, this study also experienced some limitations. Ideally, both workshops would be conducted in a face-to-face setting instead of the online setting in Belgium. This would facilitate even more creativity and sharing experiences among the participants. Moreover, recruitment challenges limited us to one workshop with two sessions in each country. Although we gained many new perspectives and ideas, more workshops with more participants would allow for saturation among the population instead of saturation among the sample. Regarding the sample of these workshops, we focused on homeowners that had measured (high) radon levels in their homes. Although this was the purpose of the study, it created selection bias.

### 5.2. Future Research

Future research should explore more participatory research designs, both in intervention design research and radon health communication, emphasizing different social categories and countries. Moreover, scholars could investigate more comprehensive communication strategies with adapted messages depending on the sample’s behavior change stage. Finally, researchers could explore the ideas provided by the participants further in terms of theoretical framework, but also in terms of effectiveness in a lab setting.

## 6. Conclusions

In this study, the questions were raised: what are the main barriers and facilitators to engaging in radon-protective behavior experienced by homeowners, and how are these addressed in current interventions? Second, how can the communication about radon be improved to be more relevant and engaging for the target group?

To investigate these questions, we designed a participatory co-design research method with homeowners in Belgium and Slovenia. The findings of these workshops show that participants require more policy and legislation, for instance, about certifying mitigation companies or including radon measurement on the energy certificate. Moreover, they experience a need for support from the state during radon testing and mitigating procedures, both in terms of financial aid and communication or advice. Furthermore, they indicated a need for more awareness among the general public and, more specifically, a lack of engagement. A holistic communication approach is also needed, including by stakeholders such as general practitioners and architects.

When looking at communication specifically, both workshops suggested that communication strategies should be amended to match the stage from awareness to having a radon-safe home. Communication tools such as radio spots with humor or personalized letters to raise awareness and engagement were proposed. Further, testimonials were pointed out as an effective way to highlight the issues and solutions of people who reported similar experiences. Further research should adopt co-design methods, both in research about radon health communication and in different fields. Further, scholars could test the effectiveness of some of these ideas in a controlled setting and in an integrated, multi-stage intervention.

## Figures and Tables

**Table 1:** Belgium: problem statements—converging.

Problem Statement	Count
How to touch (i.e., inform and raise awareness of) people?	IIII
How to guarantee positive results of the mitigation work?	IIII
How to motivate people to test their homes?	III
How to shock people to act?	III
How to facilitate the necessary steps (information, tests, mitigation companies)?	III
How to find a professional company that has experience with radon?	II
How to get help to mitigate?	II
What is the risk if I do nothing?	II
How to inform the authorities of the problem and the dangers?	II
How to find reliable information to decide on home mitigation?	II
How to motivate people, when they have a radon problem, to mitigate it?	I
How to incorporate a law which obliges to incorporate radon measurements in all new constructions?	I
How to establish an EU standard (because of different levels of what is “dangerous”)?	I

**Table 2:** Belgium: solution statements—converging.

Solution Statement	Count
Catchy radio spot (humor)	III
Raising awareness via the communities	I
Flyers for all households that summarize the problem, the consequences, and the solutions (links to more information)	I
TV news report	I
Give visibility to solutions and companies that can help (who had training on the subject and solutions)	I
A booklet: a small workbook for children	I
Workshops at building fairs	I
Integrating radon in the electricity certificate of a house	I

**Table 3:** Slovenia: problem statements—converging.

Problem Statement	Count
How to get subventions from the state?	IIIII
How to increase awareness about radon in the population?	III
How to have legal requirements for new buildings with clear guidance on technical aspects?	III
How to have more advisors (also for private dwellings)?	III
How to include the topic of radon in schools?	II
How to get access to free dosimeters?	II
How to limit the costs related to mitigation?	I
How to know what to do step-by-step after receiving the measurement result?	I
How to adopt a similar approach as the COVID-19 communication (i.e., on many levels, multidisciplinary)?	I
How to work together with all experts? And how to communicate and increase awareness about measurements, mitigation, general information about radon, and the process of mitigation, including potential subventions?	I
How to have more dosimeters per dwelling, not only one (as it is now)?	I

**Table 4:** Slovenia: solution statements awareness—converging.

Solution—Awareness	Count
Informing households by post (similar to post during elections)	IIII
Interactive portal about radon	IIII
Articles in local newspapers	III
Lectures at expert associations/organizations	II
Contribution about radon in a TV morning program	II
Regular information about radon in mass media	II
Personal testimonials of influencers	I
Include radon education in schools	I
Round table discussions and lectures at a local community	I

**Table 5:** Slovenia: solution statements advice about mitigation—converging.

Solution—Advice	Count
Personal testimonials of people during mitigation	IIII
Dedicated, specialized magazine on mitigation and industry providing mitigation works or material	III
Informing households by post	II
Lectures on the internet (YouTube), a podcast	II
National professional qualifications for radon mitigation	II
Guidance on further steps after measurements	I
More information on successful mitigation and re-tests in mass media	I
Information should include a technical explanation and also an explanation of the physics related to radon	I

## Data Availability

The data that support the findings of this study are available from the corresponding author, S.A., upon request.

## Appendix A. Homeowner Journey

**Journey Steps**	**Awareness**	**Evaluation**	**Purchase**	**Delivery**	**Action**	**Reassure**	**Ambassadorship**
	I understand radon is a threat	I perceive it as a possible risk (affect + consequence)	I want to get the threat level assessed	I get my results back	I take action to mitigate my house	I do a new test to assure I’m safe	I tell my friends and family
Homeowner thoughts/ Motivations	I’m interested to know more I’m probably not affected It’s one of many risks nowadays	I need to find out if it affects my household	Where do I need to request this? How much will it cost?	Worried or reassured confused What about my health? What’s the damage?	Determined Positive (to protect family)	I need to find out if all my efforts paid off hopeful	My friends and family need to protect themselves as well
Homeowner actions/ Jobs to be done	Noticing info Trying to find more information Reading and understanding info	Find information about testing Find info on what to do next	Gathering information Ordering test Executing the test myself	Understanding the letter Deciding to take action (or not) Go see your doctor	Gathering information Contacting companies Requesting offers Selecting a company and plan Executing mitigation Ventilate house	Order a new test Executing the test	Talking about your experience Reassuring Encouraging
Questions/Barriers	Difficult to understand the information Considering the relevance for me	What to do now? Where can I test? How much will it cost?	Cost? How to do it? How long before I get my result back?	What do these results mean? What’s next?	What to do? What will it cost? is it enough? will it help, etc.? A lot of work Is it worth it?	What if it didn’t help? Were all costs and efforts useless?	What will others think if my house was unsafe for so long? What about the value of my house?
Emotional State	Neutral	Worried	Motivated overwhelmed	Insecure, afraid/ Reassured	Motivated Overwhelmed	Insecure hopeful	Happy concerned
Touchpoints with homeowner	Press articles Government initiatives	Personal environment Government (local) Doctor (local) Google	Website Correspondence test Personal environment	Report	Architects Construction companies	Website Correspondence test Correspondence result	Newsletter Website Social media Interpersonal communication
Potential for improvement	Communication mix approach (different push channels) Testimonials	Convincing and informing via doctors	Guidance external party Digital meters Making explicit who has tested before	Guidance Providing different options in a report Providing rational arguments next to health Informing about the impact on family members (different generations)	Tailor-made solution Reassuring it was a good choice (also during the process)	Providing options on what to do if the test is not ok	Breaking the taboo Providing a social norm to mitigate Mitigating is a normal thing to do

## Appendix B. Workshop Belgium

### Appendix B.1. Session 1: Problem Definition

- How to “touch” (inform and raise awareness of) people?
- How to motivate people to test their homes?
- How to find the right solution for the specific house?
- How to be close to people to touch them? Local networks (municipalities, etc.) mix of trust/proximity
- How to find/obtain help from the state for the mitigation works?
- How to motivate people, when they have a radon problem, to mitigate it?
- How to make people aware/make the problem more tangible through press articles (popularization)?
- How to train the medical profession to properly diagnose the resulting diseases?
- How to find reliable information to decide on home mitigation?
- How to visualize the danger?
- How to find a professional company that has experience with radon/possible solutions?
- How to facilitate the necessary steps (information, texts, mitigation companies)?
- How to communicate about a seemingly hypothetical danger?
- How to inform the authorities of the problem and the dangers?
- How can companies find information to train on the subject?
- How to approach the financial aspect (mitigation works)?
- How to communicate so that it is understandable to the general population?
- How to shock people to take action?
- How to be well informed (on radon, solutions)?
- How to create a checklist/decision tree?
- How to ensure a radon evaluation standard for renovation companies?
- How to guarantee positive results of the mitigation work?
- How to know if mitigation is going to be expensive?
- How to highlight the advantages of the steps required of people?
- How to avoid “information fatigue”?
- How to encourage cross-border cooperation?
- Won’t people think I’m an overly worried alarmist?
- Do we need expert guides for mitigation projects?
- How to get help to mitigate?
- What is the risk if I do nothing?
- How to quantify the risk (compared to other dangers, to other houses in the region, etc.)?
- How to certify a company as ISO9000 but for radon?
- How to establish an EU standard (because of different levels of what is “dangerous”)?
- Am I the only one in my neighborhood, my region, to worry and act?
- Should we have a law that obliges to incorporate radon measures in all new constructions?
- How much time will I have to spend fixing the problem?

### Appendix B.2. Clustering

#### Appendix B.2.1. Installing Standardization to Ensure Quality

- How to certify a company as ISO9000 but for radon?
- How to establish an EU standard (because of different levels of what is “dangerous”)?
- Do we need expert guides for mitigation projects?
- How to ensure a radon evaluation standard for renovation companies?
- Should we have a law that obliges to incorporate radon measures in all new constructions?
- How to guarantee positive results of the mitigation work?
- How to encourage cross-border cooperation?

#### Appendix B.2.2. Clarify a Stepped Approach

- How to create a checklist/decision tree?
- How to get help to mitigate?
- How to facilitate the necessary steps (information, texts, mitigation companies)?
- How to find the right solution for the specific house?

#### Appendix B.2.3. Communication through Different Stakeholders

- How to train the medical professionals to properly diagnose the resulting diseases?
- How can companies find information to train on the subject?
- How to inform the authorities of the problem and the dangers?
- How to be close to people to touch them? Local networks (municipalities, etc.) mix of trust/proximity.

#### Appendix B.2.4. Sensitizing Steps and Thresholds

- How to make people aware/make the problem more tangible through press articles (popularization)?
- How to motivate people, when they have a radon problem, to mitigate it?
- Won’t people think I’m an overly worried alarmist?
- How much time will I have to spend fixing the problem?
- How to avoid “information fatigue”?
- Am I the only one in my neighborhood, my region, to worry and act?
- How to motivate people to test their homes?
- How much time will I have to spend fixing the problem?

#### Appendix B.2.5. Cost of Mitigation

- How to know if mitigation is going to be expensive?
- How to approach the financial aspect (mitigation works)?

#### Appendix B.2.6. Mitigation Contractors

- How to find/obtain help from the state for the mitigation works?
- How to find a professional company that has experience with radon/possible solutions?

#### Appendix B.2.7. Communication

- What is the risk if I do nothing?
- How to visualize the danger?
- How to be well informed (on radon, solutions)?
- How to highlight the advantages of the steps required of people?
- How to communicate so that it is understandable to the general population?
- How to quantify the risk (compared to other dangers, to other houses in the region, etc.)?
- How to “touch” (inform and raise awareness of) people?
- How to communicate about a seemingly hypothetical danger?
- How to shock people to take action?
- How to find reliable information to decide on home mitigation?

### Appendix B.3. Session 2: Ideas

- Advertising campaign via social media (Facebook, etc.)
- Testimonial of someone who easily mitigated
- Flyers in places of passage (waiting room, bakery, etc.)
- Catchy radio spot (possibly humor)
- Show examples of mitigation so people realize it’s doable
- Information session
- Emphasize that taking a test is cheap and easy
- Raising awareness via the Communes (Urbanism—environment)
- Press articles at the start of annual campaigns (already done)
- Raising awareness via the Fondation contre le Cancer and associations dealing with the theme of health and cancer
- Comprehensive website
- Cinema spots before the movie starts
- Create a “radon safe” label
- Go to doctors in relevant regions to raise awareness of the medical problem—flyers in waiting rooms, for example
- Flyers for all households that summarize the problem, the consequences, and the solutions/links to more information
- Advertising on media channels
- An episode on TV (peak time)
- Focus information on the “simple” feasibility of mitigation (in general)
- Commercial signs at bus stations
- Organize a competition with prizes for people who have done mitigation in the past year
- Communication is clear if it becomes a condition for the sale of a house/apartment
- TV news report
- Carry out workshops
- Training
- Make a booklet: a small workbook for children, to make them talk to their parents and raise their awareness.
- Include information on official invoices
- Integrate a radon measurement into the PEB (as for the Housing audit)
- A radon center, as we have for the cancer.pr, to find help
- Mitigation and cost communicate through flyers and more detailed by the website
- Harmonization of radon “danger” thresholds in the EU
- Target the concerned people first
- Workshops in Batibouw, etc.
- Workshops in primary schools
- Photos that show the simplicity of mitigation
- Create an entry in the yellow pages with “radon service”
- Must give more visibility to solutions and companies that can help (who have training on the subject and solutions)
- Flyer “All buildings/houses”
- Question and answer booklet
- Professionals (buildings) who on other projects addressed the problem and raised awareness
- Inform about the radon risk in building permit applications (and preventive measures)
- Emphasis on the discretion of the result

### Appendix B.4. Homeowner Journey Mapping

**Radon Awareness**	**Evaluation (Before Testing)**	**Action (i.e., Mitigation)**	**Ambassadorship**
- Advertising campaign via social media (Facebook, etc.) - Raising awareness via the communities - Flyers for all households that summarize the problem, the consequences, and the solutions/links to more information - Raising awareness via the Fondation contre le Cancer and associations dealing with the theme of health and cancer - TV news report - An episode on TV (peak time) - Make a booklet - (Small workbook for children) - Press articles at the start of annual campaigns - Catchy radio spot (possibly humor) - Go to doctors in relevant regions to raise awareness of the medical problem—flyers in waiting rooms for example - Advertising on media channels - Communication is clear if it becomes a condition for the sale of a house/apartment - Training - Include on official invoices - Workshops in primary schools - Flyer “All buildings/houses” - Professionals (buildings) who on other projects raised the problem and raised awareness - Flyers in places of passage (waiting room, bakery, etc.) - Integrate a radon measurement into the PEB (as for the Housing audit) - Inform about the radon risk in building permit applications (and preventive measures)	- Advertising campaign via social media (Facebook, etc.) - Catchy radio spot (possibly humor) - Advertising on media channels - Information session - Workshops in primary schools - Communication is clear if it becomes a condition for the sale of a house/apartment - Emphasize that taking a test is cheap and easy - Press articles at the start of annual campaigns - Comprehensive website - Carry out workshops - Make a booklet - (small workbook for children) - Question and answer booklet - Emphasis on the discretion of the result - Go to doctors in relevant regions to raise awareness of the medical problem—flyers in waiting rooms for example - Flyers for all households that summarize the problem, the consequences, and the solutions/links to more information - An episode on TV (peak time) - Mitigation and cost communicate through flyers and more detailed by the website - Workshops in Batibouw, etc. - Inform about the radon risk in building permit applications (and preventive measures) - Integrate a radon measurement into the PEB (as for the Housing audit)	- Testimonial of someone who easily mitigated - Mitigation and cost communicate through flyers and more detailed by the website - Flyers for all households that summarize the problem, the consequences, and the solutions/links to more information - Show examples of mitigation so people realize it’s doable - Create an entry in the yellow pages with “radon service” - Professionals (buildings) who on other projects raised the problem and raised awareness - Integrate a radon measurement into the PEB (as for the Housing audit) - Comprehensive website - An episode on TV (peak time) - Focus information on the “simple” feasibility of mitigation (in general) - Communication is clear if it becomes a condition for the sale of a house/apartment - Organize a competition with prizes for people who have done mitigation in the past year - Must give more visibility to solutions and companies that can help (who have training on the subject and solutions) - Photos that show the simplicity of mitigation	- Advertising campaign via social media (Facebook, etc.) - Testimonial of someone who easily mitigated - Create a “radon safe” label - Organize a competition with prizes for people who have done mitigation in the past year - TV news report

## Appendix C. Workshop 2: Slovenia

### Appendix C.1. Session 1: Problem Definition

- Understandable and accessible information about mitigation.
- There is too little comprehensive information available on the national level.
- We need to give more information to residents. Those information needs to be constant and not in intervals (not in short information campaigns). There is an information gap between campaigns.
- We need to increase awareness of young people in schools. More information is needed about mitigation. More help/advice is needed about how to address the exceeded levels of radon in a dwelling.
- We need to include the topic of radon in schools.
- We need to increase awareness about radon in the population.
- To inform and make people aware of radon risks.
- We need to take the same approach as was applied in COVID-19 communication (on many levels, multidisciplinary, intense, etc.).
- All newly built documentation should contain radon-related information.
- Media need to stop spreading negative information about radon (e.g., that we don’t measure it enough), but rather focus on actions—what can we do?
- That media would report also on the results of measurements.
- I wish that all experts, all scientists regardless of disciplines would work together (physicians, builders, physics, etc.).
- All experts need to work together and they need to communicate and increase awareness about measurements, mitigation, general information about radon, about the process including potential subventions.
- We wish to have the possibility to borrow an active dosimeter.
- We wish to have better access to dosimeters (passive and active).
- Accessible, free dosimeters.
- Enough dosimeters.
- To have more dosimeters per dwelling, not only one as it is now.
- To make free measurements available to all residents.
- To have active dosimeters available, to be able to follow radon concertation at any time.
- To know where to get active dosimeters.
- That an expert would come to measure radon.
- After receiving the results of a measurement, I didn’t get any information on contractors for mitigation.
- To have somebody to advise what to do after measurements.
- Would need to know what to do step-by-step after receiving the measurement result. Who can help me?
- That somebody would guarantee the quality of the mitigation work.
- How to do mitigation of an old building?
- I got an answer that those experts on a national level advise only on public buildings and not on private dwellings.
- That state would support also private dwellings and not only public buildings.
- To have advice on mitigation also for individual dwellings.
- To have more advisors.
- More control by the inspectorate.
- To have more mitigation services available.
- The costs related to mitigation would not be too high.
- To know how much mitigation would cost.
- Subventions.
- To get subventions from the state.
- Will (would) the state co-finance the mitigation?
- Protection of radiation in a new build.
- It should be obligatory for a new build to integrate a protective layer (foil).
- To have a legal requirement for a new build with clear guidance on technical aspects.
- There is no need to change the legal act. We need to implement it.
- The ICRP-65 is still used for dose calculation. Authorities postpone the implementation of ICRP-137 yearly.
- Radon information should be part of an energy certificate.
- Positively encourage employers to do the measurements and not only to point out what is not done.

### Appendix C.2. Clustering

#### Appendix C.2.1. Communication, Information, Awareness

- Understandable and accessible information about mitigation.
- There is too little comprehensive information available on the national level.
- We need to give more information to residents. This information needs to be constant and not in intervals (not in short information campaigns). There is an information gap between campaigns.
- We need to increase awareness of young people in schools. More information is needed about mitigation. More help/advice is needed about how to address the exceeded levels of radon in a dwelling.
- We need to include the topic of radon in schools.
- We need to increase awareness about radon in the population.
- To inform and make people aware of radon risks.
- All newly built documentation should contain radon-related information.
- Media need to stop spreading negative information about radon (e.g., that we don’t measure it enough), but rather focus on actions—what can we do?
- That media would report also on the results of measurements.

#### Appendix C.2.2. Advice after Measurement

- After receiving the results of a measurement, I didn’t get any information on contractors for mitigation.
- To have somebody to advise what to do after measurements.
- Would need to know what to do step-by-step after receiving the measurement result. Who can help me?
- To have advice on mitigation also for individual dwellings.
- To have more advisors.
- I got an answer that those experts on a national level advise only on public buildings and not on private dwellings.

#### Appendix C.2.3. Comprehensive—Holistic Approach

- I wish that all experts, all scientists regardless of disciplines would work together (physicians, builders, physics, etc.).
- We need to take the same approach as was applied in COVID-19 communication (on many levels, multidisciplinary, intense, etc.).
- All experts need to work together and they need to communicate and increase awareness about measurements, mitigation, general information about radon, about the process including potential subventions.

#### Appendix C.2.4. Accessibility for Passive and Active Dosimeters and Measurement Support

- We wish to have the possibility to borrow an active dosimeter.
- We wish to have better access to dosimeters (passive and active).
- Accessible, free dosimeters.
- Enough dosimeters.
- To have more dosimeters per dwelling, not only one as it is now.
- To make free measurements available to all residents.
- To have active dosimeters available, to be able to follow radon concertation at any time.
- To know where to get active dosimeters.
- That an expert would come to measure radon.

#### Appendix C.2.5. Mitigation Support

- That somebody would guarantee the quality of the mitigation work.
- How to do mitigation of an old building?
- That state would support also private dwellings and not only public buildings.
- More control by the inspectorate.
- To have more mitigation services available.

#### Appendix C.2.6. The Financial Burden of Mitigation

- The costs related to mitigation would not be too high.
- To know how much mitigation would cost.
- Subventions.
- To get subventions from the state.
- Will (would) the state co-finance the mitigation?

#### Appendix C.2.7. Legal Requirement

- Protection of radiation in a new build.
- It should be obligatory for a new build to integrate a protective layer (foil).
- To have a legal requirement for a new build with clear guidance on technical aspects.
- There is no need to change the legal act. We need to implement it.
- The ICRP-65 is still used for dose calculation. Authorities postpone the implementation of ICRP-137 yearly.
- Radon information should be part of an energy certificate.

#### Appendix C.2.8. Motivation

- Positively encourage employers to do the measurements and not only to point out what is not done.

### Appendix C.3. Session 2: Ideas (Awareness)

- Advertisements on YouTube (sponsored advertisements);
- Inform teachers in primary and secondary schools on opportunities to make small study-practical projects related to radon measurements and radon status in the region. This should be connected with Radon Action Plan;
- Tik-Tok and Instagram for the younger population;
- Round table discussions and lectures about radon given by experts in their local community;
- Popular articles in specific journals, for instance, in the journal Jana (Jana is a popular weekly women’s magazine discussing healthy lifestyles and celebrities);
- Regular information about radon in mass media;
- Interactive portal about radon and a lot of radon information established and managed by authorities responsible for radon management;
- Include radon and its risks in school competitions such as “Happy school” (Vesela sola—it is the most known yearly national inter-school competition in different categories according to scientific disciplines. All Slovenian schools are included in the competitions which are organized bottom-up, from local, regional, and national levels);
- Contributions about radon in the TV program “Good morning” (Dobro jutro is a daily morning program, talk show on national TV);
- Lectures about radon at GZS (Slovenian Chamber of Commerce) and OZS (Chamber of Commerce and Industry of Slovenia);
- Inclusion of radon in options to be selected for chemistry and biology in secondary schools;
- Inclusion of radon in a TV program, such as “Bite a science” (Ugriznimo znanost is a popular science program on the national RTV);
- News articles in local newspapers;
- A communication intervention: First, a questionnaire should be conducted to assess what the resident knows about radon, whether the resident is afraid of it, etc. Second, the type of communication and personal advisor is selected based on the results from the questionnaire. When the advisor finds out what the person with radon in the house doesn’t understand, they provide explanation. It is important to give a feeling that is not shameful not to know those things, the advisor needs to be empathic. Third, the advisor asks more questions and sends a video responding to the questions. The advisor should explain in a dialogue that there are many solutions for the radon problem available;
- Testimonials of influencers;
- Personal letter to all households (such as for elections);
- General information about radon (what is radon, what are risks, etc.);
- The state has to inform which areas are under radon risk;
- More publications about radon in mass media;
- Scientific publications in scientific journals (there is a lack of knowledge about radon also among experts);
- Publication of news articles in local mass media;
- Publish results of measurements in local media.

### Appendix C.4. Session 2: Ideas (Advice)

- We should explain how to remove radon from a dwelling with physical and technical information;
- Personal communication with a selected advisor who is respectful and gives realistic information on mitigation;
- Inform households by post;
- Tik-Tok, Instagram;
- YouTube advertisement;
- National professional qualification for radon mitigation;
- Lectures on the internet (YouTube), Podcast;
- More information on successful mitigation and re-tests in mass media;
- Guidance on further steps after measurements;
- Have a list of mitigation contractors;
- Explanation on how to understand results;
- Personal testimonials of people during mitigation;
- Dedicated, specialized magazine on mitigation and industry providing mitigation works or material.
